# Two decades of implantable cardioverter defibrillator implantation and follow-up at a South African referral centre: trends, indications and long-term outcomes in a resource-limited setting

**DOI:** 10.1186/s42444-022-00070-2

**Published:** 2022-08-01

**Authors:** Philasande Mkoko, Kayla Solomon, Ashley Chin

**Affiliations:** 1 Division of Cardiology, Department of Medicine, Faculty of Health Sciences, The University of Cape Town, Cape Town, South Africa; 2 E17 Cardiac Clinic, Groote Schuur Hospital, Cape Town, South Africa

## Abstract

**Background:**

More than two-thirds of cardiovascular deaths occur in low- and middle-income countries. Sudden cardiac deaths (SCD) from ventricular arrhythmias are an important cause of cardiovascular deaths. Implantable cardioverter defibrillators (ICD) are an important therapeutic strategy for detecting and terminating ventricular arrhythmias in patients at risk of SCD. The profile of patients treated with ICDs in South Africa is unknown. Further, with changing lines of evidence, the implantation trends are undetermined. The objectives of this study were to determine the profile of ICD recipients and implantation trends in a South African quaternary hospital.

**Methods:**

This was a retrospective review of all patients implanted with ICDs at Groote Schuur Hospital from 01 January 1998 to 31 December 2020. A standardised data collection form was used to collect baseline demographic data, information on clinical presentation and ICD follow-up data for the history of ICD shock therapies.

**Results:**

A total of 253 ICDs were implanted; 75% for secondary prevention and 25% for primary prevention. 67.2% of the implanted ICDs were single-chamber ICDs, dual-chamber ICDs were implanted in 12.3% and Cardiac resynchronisation with a defibrillator (CRT-D) in 20.6%. There was an upward trajectory of ICD implantations during the study period. Increasing numbers of dual-chamber devices and CRT-D were implanted over time. ICD recipients had a mean (standard deviation) age of 50 (14) years and were predominantly male (69%). Primary prevention ICD recipients were younger than secondary prevention recipients, with a mean (SD) age of 46 (14) years versus 51 (14) years, *p* = 0.019. The secondary prevention group presented with ventricular tachycardia in 81%, ventricular fibrillation in 13% and cardiopulmonary resuscitation without documented heart rhythm in 5.3% (10/190). After a median (interquartile range) follow-up of 44 (15; 93) months, there was an overall mortality rate of 16.2%, with no mortality difference between the primary and secondary prevention patient groups.

**Conclusion:**

There is an increase in the annual number of ICDs implanted at a South African referral centre. ICDs are predominantly implanted for secondary prevention. However, over time the number of devices implanted for primary prevention is steadily increased. During follow-up, there was no mortality difference between the primary prevention and the secondary prevention groups.

## Background

Cardiovascular diseases are a significant public health problem. Globally, at least 17 million lives are lost due to cardiovascular disease each year, and more than 75% of these deaths occur in low- and middle-income countries [1]. Sudden cardiac death (SCD) is a leading cause of cardiovascular death. In the developed nations, SCD accounts for about 50% of all cardiovascular deaths [2, 3], 25% of these being a first symptomatic cardiac event [2, 4, 5]. In Europe and North America, SCD accounts for approximately 350,000 deaths per year [4, 6–8]. Ventricular arrhythmias are the major cause of SCD. For example, in 157 patients who suffered SCD while wearing a Holter monitor, Bayes de Luna and colleagues reported that 84% of patients had Ventricular Tachycardia (VT) or Ventricular Fibrillation (VF), and 16% had a bradyarrhythmia as the cause of SCD [9]. This finding has been corroborated by more contemporary data [10]. Implantable cardioverter defibrillators (ICD) are an established therapeutic intervention for terminating VTs and VF in at-risk patients. Currently, implantation of ICDs for primary prevention and secondary prevention in at-risk patients is supported by current guidelines [11, 12].

There are increases in ICD implantations in Europe and North America [13–15]. However, there are limited data on implantation trends and long-term outcomes of patients receiving such treatment in resource constraint settings like South Africa. The objectives of this study were to determine the trends of ICD implantations and the long-term outcomes of ICD recipients in a South African referral centre.

## Methods

### Study design

This study was designed to review all patients implanted with ICDs at Groote Schuur Hospital (GSH) from 01 January 1998 to 31 December 2020. Groote Schuur Hospital is a 900-bed tertiary and quaternary care centre located in the Western Cape province of South Africa and affiliated with the University of Cape Town (UCT).

### Data collection

All patients implanted with ICDs are followed up at the GSH device clinic six weeks post-implantation with a clinical review, a chest radiograph, an electrocardiogram (ECG), and device interrogation. The clinical examination, ECG and device interrogation are repeated every six months. Patients with a CRT-D perform a 6-min walk on each visit. Patients are advised to report at the device clinic when they experience an ICD shock. Device stored electrograms (EGMs) are reviewed by the electrophysiologist to determine whether the delivered shock therapy was appropriate or inappropriate and to attempt to elucidate the cause of inappropriate ICD discharge when it was not appropriate.

Clinical notes, ICD device information, and follow-up data were reviewed. A standardised data collection form was used to collect baseline demographic data, information on clinical presentation and ICD follow-up data for the history of ICD shock therapies. This study was approved by the University of Cape Town Human Research Ethics Committee (UCT HREC REF: 505/2019).

### Statistical analysis

Normally distributed continuous variables are reported as means [standard deviations (SD)] and as medians [interquartile ranges (IQR)] when skewed. Discrete data are presented as numbers and percentages. The Chi-square test and the Student’s *T* test were used to calculate differences between the primary prevention and the secondary prevention groups accordingly. The Kaplan–Meier and log-rank tests assessed the cumulative survival differences between the primary prevention group versus secondary prevention. A *p* value < 0.05 represents a statistically significant difference. Statistical analyses were performed using SPSS Statistics for Macintosh version 24.0 (IBM, USA).

## Results

Between 1998 and 2020, 253 ICDs were implanted at Groote Schuur Hospital, including 179 (75%) ICDs implanted for secondary prevention and 63 (25%) for primary prevention. Single chamber ICDs were implanted in 170 (67.2%), Dual-chamber ICD in 31 (12.3%), Cardiac Resynchronisation with a defibrillator (CRT-D) in 52 (20.6%) and 9 (3.6%) CRT-D upgrades from single-chamber ICDs. The single-chamber ICDs were predominantly implanted in the secondary prevention patient population and the CRT-D in the primary prevention group. The patient characteristics of ICD recipients are presented in Table 1. The mean (SD) age at ICD implantation was 50 (14) years. The primary prevention patient population was younger than the secondary prevention patient population with a mean age (SD) of 46 (14) years versus 51 (14) years, *p* = 0.019. Males accounted for 69% of the overall patient population, with no differences between the primary prevention group and the secondary prevention group regarding gender distribution. Regarding baseline comorbidities, baseline ischemic heart disease was present in 44% of the secondary prevention group versus 24% in the primary prevention group, *p* = 0.009. Patients receiving primary prevention were predominantly receiving adequate available heart failure medical therapy. For example, 92.1% were on beta-blockers, and 84.1% were on angiotensin-converting enzyme inhibitors (ACE) or angiotensin receptor blockers (ARB) before ICD implantation. Patients receiving secondary prevention devices presented with ventricular tachycardia in 155/190 (81%), ventricular fibrillation in 25/190 (13%) and cardiopulmonary resuscitation without a documented ECG rhythm in 10/190 (5.3%) (Table 2).

**Table 1:** Patient characteristics

Variable	Overall population, no: 253	Secondary prevention, no: 190	Primary prevention, no: 63	*P* value
Age, mean (SD) years	50.2 (14.7)	51.5 (14.5)	46.5 (14.8)	0.019
Male gender, No (%)	175 (69.2)	131 (68.9)	44 (69.8)	0.894
Systemic hypertension, No (%)	124 (49.0)	94 (49.5)	30 (47.6)	0.913
Diabetes mellitus, No (%)	51 (20.2)	40 (21.2)	11 (18.0)	0.730
Dyslipidemia, No (%)	45 (17.8)	36 (19.0)	9 (14.8)	0.571
Chronic Obstructive Pulmonary Disease	9 (3.6)	6 (3.2)	3 (4.9)	0.816
Ischemic Heart Disease	99 (39.1)	84 (44.4)	15 (24.6)	0.009
Atrial fibrillation/atrial flutter	22 (8.7)	18 (9.5)	4 (6.3)	0.614
LVEDd, mean (SD) (mm)	58.7 (13.2)	55.8 (11.6)	67.3 (14.1))	< 0.0001
LVESd, mean (SD) (mm)	47.5 (16.2)	44.1 (13.8)	55.5 (18.7)	< 0.0001
Ejection Fraction, mean (SD) %	36.6 (19.5)	39.8 (18.3)	27.5 (20.2)	< 0.0001
NYHA Functional Class Ι/ΙΙ, No (%)	188 (74.3)	154 (81.1)	34 (54.0)	< 0.0001
NYHA Functional Class ΙΙΙ/ΙV, No (%)	65 (25.7)	36 (18.9)	29 (46.0)	< 0.001
Statin	120 (47.4)	96 (50.8)	24 (38.1)	0.109
Beta blocker, No (%)	216 (85.4)	158 (83.6)	58 (92.1)	0.146
NDP CCB, No (%)	5 (2.0)	3 (1.6)	2 (3.2)	0.799
Amiodarone, No (%)	83 (32.8)	77 (41.0)	6 (9.5)	< 0.0001
Sotalol, No (%)	6 (2.4)	5 (2.7)	1 (1.6)	0.995
ACE-inhibitor/ ARB, No (%)	176 (69.6)	123 (65.1)	53 (84.1)	0.007
Warfarin, No (%)	52 (20.6)	35 (18.5)	17 (27.0)	0.159

*SD* standard deviation, *LVEDd* left ventricular end diastolic dimension, *LVESd* left ventricular end systolic dimension, *NDP CCB* non-dihydropyridine calcium channel blocker, *ACE* angiotensin-converting enzyme, *IQR* interquartile range

**Table 2:** Device characteristics, indications and outcomes

Variable	Overall patient population no: 253	Secondary prevention no: 190	Primary prevention no: 63	*P* value
*Arrhythmic indication for ICD*				
Ventricular Tachycardia	155 (61.3)	155 (81.6)	0	< 0.0001
Ventricular Fibrillation	25 (9.9)	25 (13.2)	0	0.001
CPR, No documented arrhythmia	10 (4.0)	10 (5.3)	0	0.146
*Type of ICD implanted*				
Single Chamber ICD	170 (67.2)	158 (83.2)	12 (19.0)	< 0.0001
Dual Chamber ICD	31 (12.3)	20 (10.5)	11 (17.5)	0.218
CRT-D	52 (20.6)	12 (6.3)	40 (63.5)	< 0.0001
CRT D upgrade from single chamber ICD	9 (3.6)	8 (4.2)	1 (1.6)	0.561
*Primary cardiac diagnoses*				
Ischemic Cardiomyopathy	73 (28.9)	61 (32.1)	12 (19.0)	0.068
Idiopathic Dilated Cardiomyopathy	65 (25.7)	30 (15.8)	35 (55.6)	< 0.0001
Peripartum Cardiomyopathy	7 (2.8)	2 (1.1)	5 (7.9)	0.015
Hypertrophic Cardiomyopathy	10 (4.0)	7 (3.7)	3 (4.8)	0.994
ARVC	32 (12.6)	29 (15.3)	3 (4.8)	0.051
Cardiac Sarcoidosis	10 (4.0)	7 (3.7)	3 (4.8)	0.994
Long QT	3 (1.2)	3 (1.6)	0	0.740
Brugada Syndrome	2 (0.8)	1 (05)	1 (1.6)	1.000
Surgically repaired CHD, No (%)	11 (4.3)	11 (5.8)	0	0.117
No Primary diagnoses made	44 (17.4)	42 (22.1)	2 (3.2)	0.001
Appropriate ICD shock	78 (30.8)	64 (33.7)	14 (22.2)	0.121
Inappropriate ICD shock	32 (12.6)	28 (14.7)	4 (6.3)	0.129
Months of Follow up, Median (IQR)	44 (25; 93)	49 (15; 94)	39 (14; 84)	0.864
Mortality, No (%)	41 (16.2)	31 (16.3)	10 (15.9)	1.000

*ARVC* arrhythmogenic right ventricular cardiomyopathy, *ICD* implantable cardioverter defibrillator, *CRT-D* cardiac resynchronization therapy plus a defibrillator, *CPR* cardiopulmonary resuscitation, *CHD* congenital heart defects

**Fig. 1:**
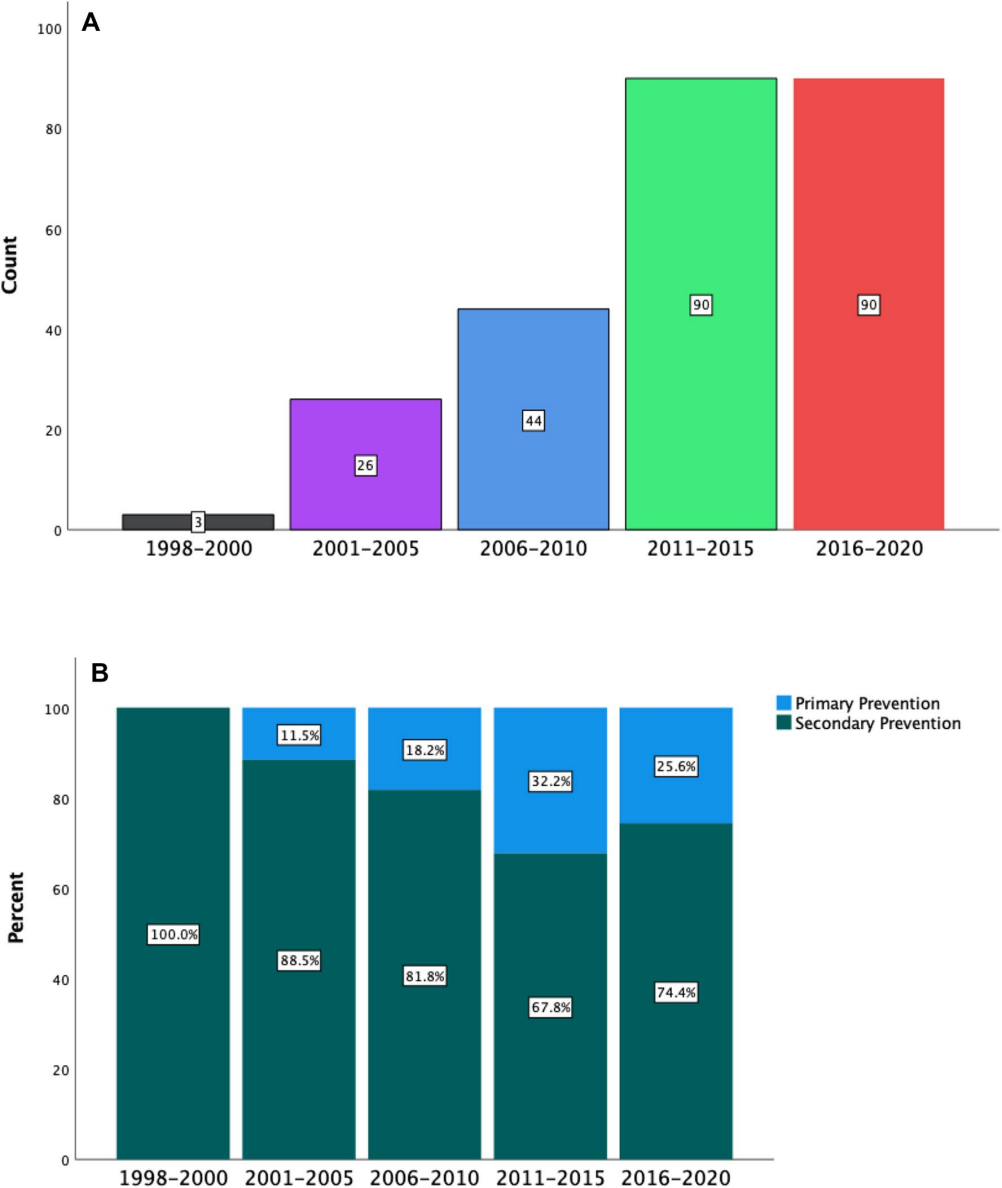
**A** Bar graph depicting the overall ICD implantation trends from 1998 to 2020. **B** Bar graph depicting increasing proportions of primary prevention ICD implants from 1998 to 2020. No = 253

**Fig. 2:**
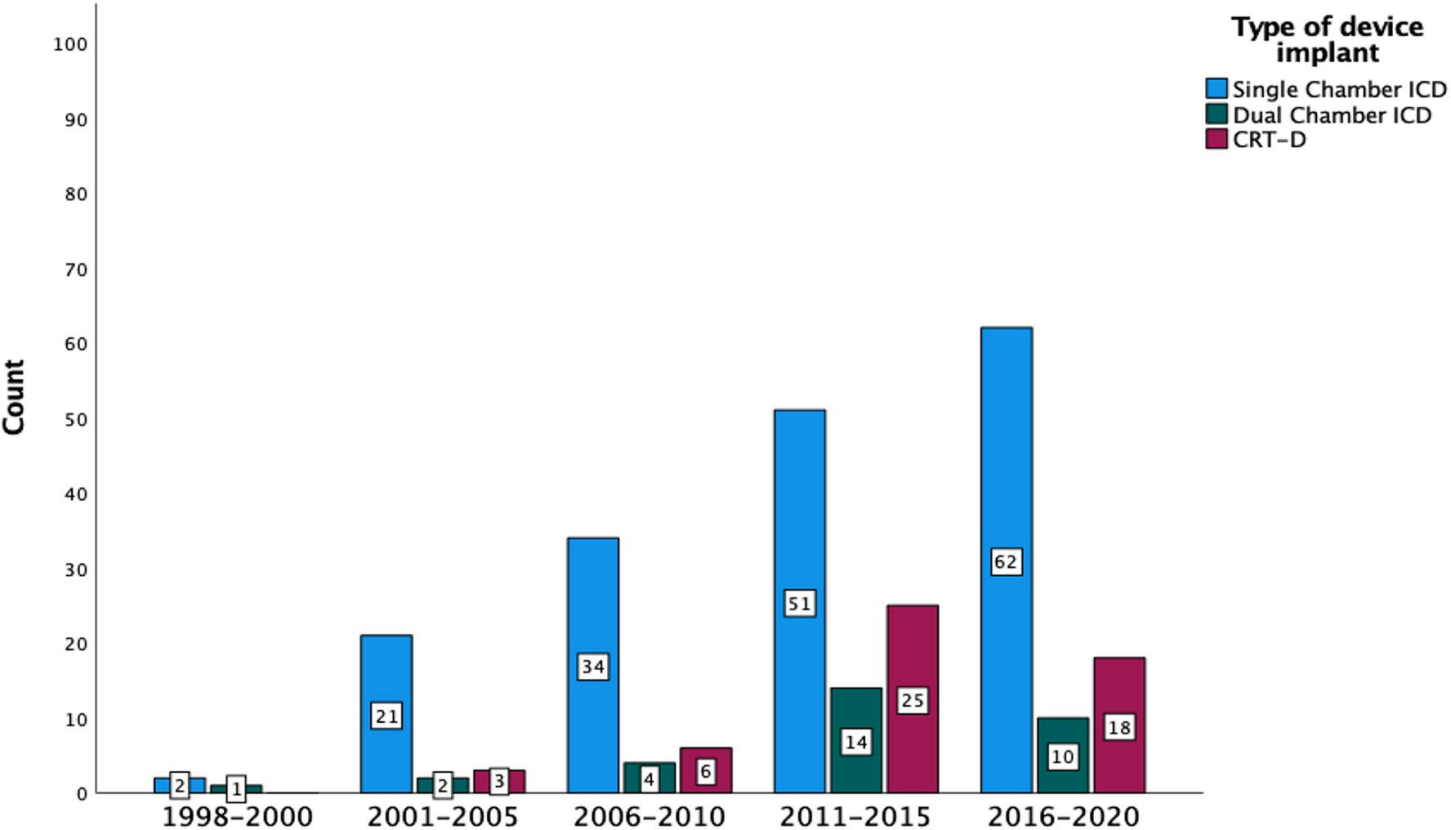
Stalked Bar Chart demonstrating an increase in implantation of Dual-chamber ICD and CRT-D on the background of an overall rise in single-chamber devices. No = 253

There is an overall upwards trend in ICD implantation in our hospital during the study period (Fig. 1A). The proportion of ICDs implanted for primary prevention increased over time. For example, there were no primary prevention devices implanted in 1998–2000 versus 11.5% in 2001–2005 versus 18.2% in 2006–2010 (Fig. 1B). Further, there was a modest increase in the implantation of dual-chamber ICDs and CRT-Ds during the study period (Fig. 2).

**Fig. 3:**
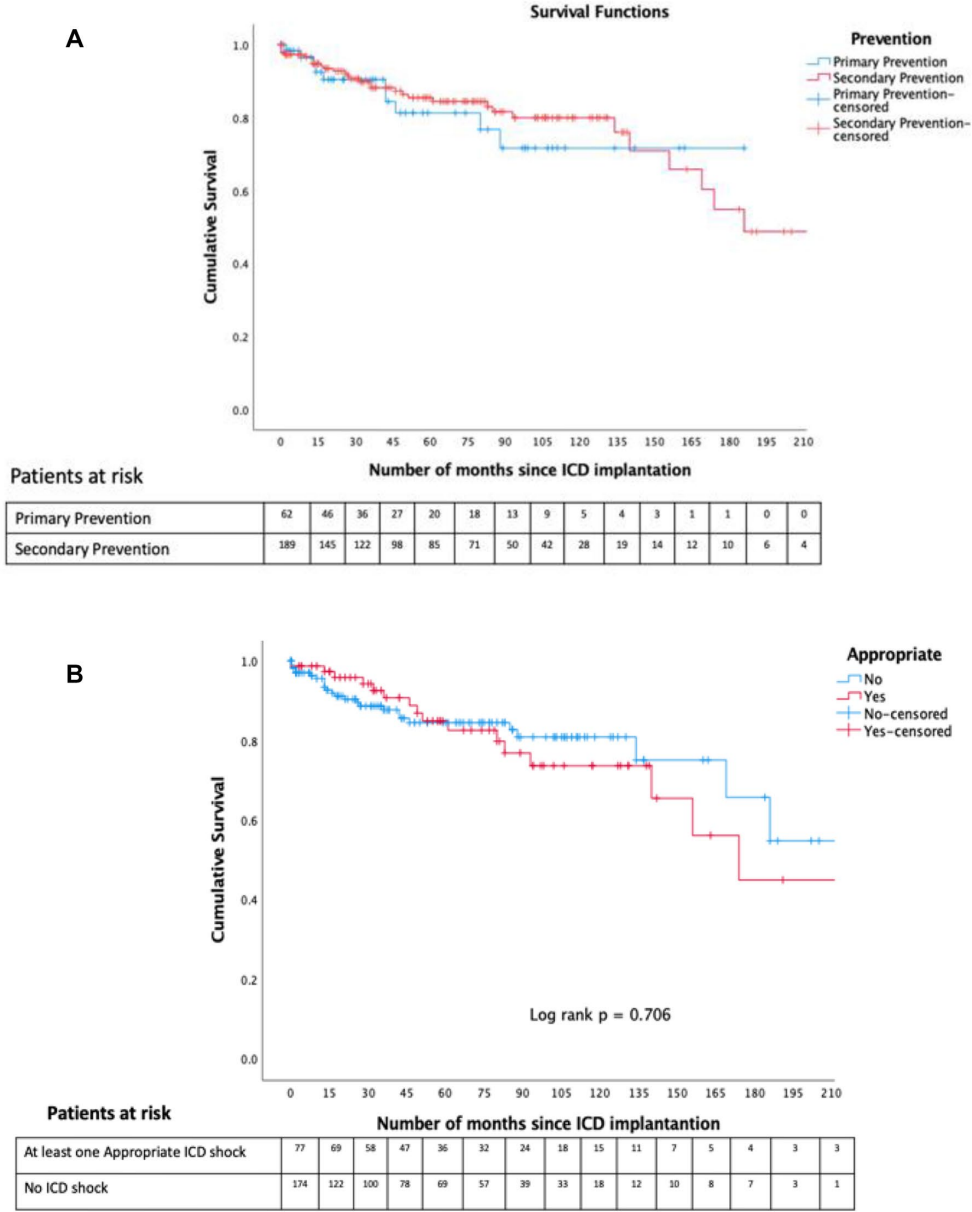
**A** Kaplan Meier curves demonstrating cumulative survival difference between patients with primary prevention and secondary prevention devices. **B** Kaplan Meier curves demonstrate survival difference between patients who received at least one appropriate ICD shock during follow versus no ICD shocks

After a median (IQR) follow-up of 44 (15; 93) months, 78 (30%) patients received at least one appropriate ICD shock, with no statistical difference between the primary prevention group and secondary prevention group, 33.7% versus 22.2%, respectively. During the follow-up period, there was an overall mortality rate of 16.2%; 16.3% in the secondary prevention group and 15.9% in the primary prevention group *p* = 1.0 (log-rank 0.682). Furthermore, there was no mortality difference between those who experienced at least one appropriate ICD shock and those who did not, log-rank *p* = 0.706 (Fig. 3).

## Discussion

The main findings of this study are that

1. This South African referral centre demonstrates an overall upward trajectory in ICD implantations.
2. Although secondary prevention ICDs predominate, the proportion of ICDs implanted for primary prevention increases over time.
3. Most of the implanted devices are single-chamber ICDs, but there have been increasing implantation rates of dual-chamber ICDs and CRT-Ds during the study period.
4. The recipients of ICDs in this South African study were younger than those reported in landmark studies and real-world data from Europe and North America.
5. There was a 16% mortality rate during follow-up with no difference between primary prevention and secondary prevention groups or those who received at least one appropriate ICD shock versus patients who did not get any ICD shock therapies.

Ventricular arrhythmias are important causes of death in patients who die suddenly while receiving cardiac monitoring with a Holter [9, 10]. In landmark primary and secondary prevention trials, ICDs have demonstrated significant mortality benefits [16–20]. Primary prevention benefits are particularly pronounced in patients with ischemic cardiomyopathy [21, 22]. There are at least modest and controversial benefits of primary prevention ICD in patients with non-ischemic dilated cardiomyopathy receiving current guideline-directed therapy [20, 21]. Because of cost implications, the uptake of ICDs in low- and middle-income countries has been slow [23–25]. This is mainly related to the device costs and a limited number of well-trained physicians to implant and follow up the patients’ [23, 24].

There is has been an upsurge in ICD implantations at Groote Schuur Hospital from 1998 to 2020. These data are consistent with ICD implantation data from North America and Europe, which demonstrated rising trends of ICD implantation [14, 15, 26, 27]. For example, in a nationwide administrative database analysis to assess the incidence of permanent pacemaker, CRT and ICD implantations in the USA between 1997 and 2004, Zhan and colleagues demonstrated a 60% increase in ICD implantations during the study period [15]. The overall implantation rates in the last term of our study, which covers the period from 2016 to 2020, were the same as those from 2011 to 2015. In the last term, this less than expected implantation rate was due to the reduction in implantations in 2019 and 2020 secondary to the coronavirus disease 2019 (COVID-19) pandemic.

Our study also demonstrated an increase in the proportion of Dual-chamber ICDs, and CRT-D implanted over time. Although there are theoretical benefits of dual-chamber ICDs over single-chamber ICD, such as improved arrhythmia detection and reduction in inappropriate ICD shocks with the implantation of the atrial lead [28], to date, these benefits have not been supported by randomised control trial data. Instead, multiple extensive nonrandomised studies have indicated that implanting Dual-chamber ICDs is associated with increased complication rates without reducing inappropriate ICD shocks or mortality compared to single-chamber ICDs [29–31]. For example, according to the US National Cardiovascular Data Registry’s (NCDR) ICD registry from 2006 to 2009, 62% of the implanted primary prevention ICDs in patients without pacing requirements were dual-chamber, and 38% were single-chamber devices [29]. The propensity-matched cohort showed no benefits of dual-chamber ICDs over single-chamber ICDs [29]. Recently, the Défibrillateur Automatique Implantable–Prévention Primaire (DAI-PP) study demonstrated that Dual-chamber ICD implantations were associated with a procedural complication rate of 12.1% versus 8.8% for single-chamber ICDs (*p* = 0.008); and over a mean follow-up of three years, pulse generators were replaced in 21.9% of dual-chamber ICD versus 13.6% of single-chamber ICD (*p* < 0.0001)[31]. Therefore considering the cost implication of Dual-chamber ICDs in patients without pacing requirements and the lack of discernible benefit, this practice is not justifiable in resource constraint settings like South Africa.

The ICD recipients in this study were younger, with an overall mean age of 50.2 ± 14.7 years, and a significant difference between the primary prevention (46.5 ± 14.8) and the secondary prevention (51.5 ± 14.5) groups, *p* = 0.019. Our patient population is younger than that presented in pivotal secondary and primary prevention ICD trials. For example, concerning secondary prevention trials, the mean age in AVID was 65 ± 11 years, in CASH, it was 58 ± 11 years, and in CIDS, it was 63.8 ± 9.9 years [16–19]. Similarly, the median (IQR) age in SCD-HeFT, a primary prevention trial, was 60.1 (51.9;69.2) years [32]. These age differences are perhaps due to the restricted access to ICDs in our setting and, therefore, the selection of younger patients with fewer comorbidities. Further, landmark primary prevention trials like the Multicenter Automatic Defibrillator Implantation Trial (MADIT), the Multicenter Unsustained Tachycardia Trial (MUSTT) and the second Multicenter Automatic Defibrillator Implantation Trial (MADIT II) exclusively included patients with established coronary artery disease. For example, 58% and 56% of the treatment group in MADIT II and MUSTT had prior coronary artery bypass grafting (CABG), respectively [22, 33]. In contrast, heart failure in Sub-Saharan Africa has been described as a disease of the young. For example, in a recent literature review and meta-analysis to describe the prevalence, aetiology, treatment and prognosis of heart failure in Sab-Saharan Africa, the mean ages of the studied patients ranged between 36.5 and 61.5 years [34]. Secondly, the common causes of heart failure in SSA, like rheumatic heart disease and peripartum cardiomyopathies, are present at a younger age [35]. Lastly, in a combined data from 12 hospital-based case series conducted between 1957 and 2005 involving 4549 patients from eight countries (Cameroon, Ghana, Kenya, Nigeria, Senegal, South Africa, Uganda and Zimbabwe), Rheumatic heart disease accounted for 22% of heart failure cases, cardiomyopathies for 20%, hypertensive heart disease for 23% and coronary artery disease accounted for only 2% of cases [35]. In the current study, idiopathic dilated cardiomyopathies accounted for the majority (55%) of the primary prevention patient population.

We further demonstrated a gender disparity in ICD implantations, with 69% of the recipients being males. This gender disparity in ICD implantation is similar to that presented in clinical trials [16–21]. This disparity is likely explained by epidemiological, clinical factors and perhaps physicians’ under-recognition of SCD risks in women [36]. For example, the lifetime risk for SCD in the Framingham heart study was 10.9% for men and 2.8% for women [37]. Further, in the Oregon Sudden Unexpected Death Study (Ore-SUDS), women were less likely than men to present with structural heart disease before sudden cardiac arrest, women had a higher prevalence of pulseless electrical activity (PEA) or asystole than that of VT or VF [38, 39]. Because only 6% of patients presenting with PEA survived to hospital discharge versus 25% of those presenting with a VT/VF [38], patients presenting with PEA are less likely to receive an ICD. Lastly, in an extensive US Nationwide Inpatient Sample database of patients suffering cardiac arrest, Kim and colleagues demonstrated that women were less likely to undergo therapeutic procedures like coronary angiography, percutaneous coronary interventions and or therapeutic temperature management [40].

In the secondary prevention group, ischemic heart disease accounted for the majority of cases (32.1%), followed by idiopathic dilated cardiomyopathy (15.8%) and arrhythmogenic right ventricular cardiomyopathy (15.3%). Indeed, the proportion of genetic cardiomyopathies was low. However, there was 22.1% of the patient population with an unknown aetiology for their sudden cardiac death. Although access to advanced cardiac imaging cardiac magnetic resonance imaging (CMR) in addition to echocardiography is available in our centre and therefore the diagnosis of structural overt arrhythmogenic cardiomyopathies like hypertrophic cardiomyopathies, arrhythmogenic right ventricular cardiomyopathy and so forth is not curtailed, we do not perform genetic testing or provocation testing to unmask possible concealed channelopathies. Therefore, a proportion of 22.1% of the secondary prevention patient population with an unknown aetiology could represent missed channelopathies or other primary electrical disorders.

## Limitations

The limitations of this study are its retrospective and single-centre design. Therefore, our findings are not necessarily representative of practice in South Africa; where there are important regional (provincial) differences to access to health care and between the private sector and public sector. For example, there are 19 electrophysiologists in South Africa, only two cardiac electrophysiologists in the public sector, and they are both in the Western Cape Province. Further, we do not have implantation-related complications due to the unavailability of these data.

## Conclusion

There has been a steady rise in ICD implantation rates at Groote Schuur Hospital between 1998 and 2020. The ICD recipients in our institution are younger than those presented in pivotal studies, and there is a gender disparity favouring male patients.

## Data Availability

Data are available from the authors upon reasonable request.

## Abbreviations

ARVC: Arrhythmogenic Right Ventricular Cardiomyopathy
ICD: Implantable Cardioverter Defibrillator
CRT-D: Cardiac Resynchronization Therapy plus a Defibrillator
CPR: Cardiopulmonary Resuscitation
CHD: Congenital Heart Defects
SD: Standard Deviation
LVEDd: Left Ventricular End Diastolic Dimension
LVESd: Left Ventricular End Systolic Dimension
NDP CCB: Non-Dihydropyridine Calcium Channel Blocker
ACE: Angiotensin-Converting Enzyme
IQR: Interquartile Range
